# Qi Fang: Affective interweave with patients

**DOI:** 10.1007/s13238-019-00667-9

**Published:** 2019-11-02

**Authors:** Tanping Fu, Wenli Duan, Mingyan Chen, Shuyang Zhang

**Affiliations:** Peking Union Medical College Hospital, Beijing, 100730 China

“It is terribly wrong to make patients serve doctors, which means they are treated as sources of medical experience. Doctors should treat patients equally. Less educated and inarticulate patients need more help, care and love. Patients’ interest is always the priority, including, not to abuse doctors’ power for personal gains, or to gain anything directly or indirectly from patients. To win a patient’s trust, we have to understand him or her, his or her understanding about the illness.” Professor Qi Fang (方圻) always repeated those views, which have profound implications (Fang, 2002).

**Figure 1:**
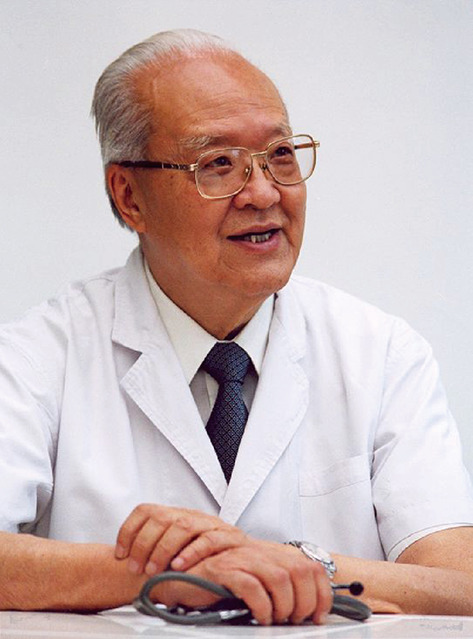
**Professor Qi Fang (1920–2018)**

Prof. Fang (1920–2018) has a distinguished career as member of Communist Party of China. He is a pioneer in the field of Chinese cardiovascular disease, First-Class Professor, honorary president of PUMCH, and representative of the 13th National Congress of the Communist Party of China (Chen et al., 2018) (Fig. 1).

**Figure 2:**
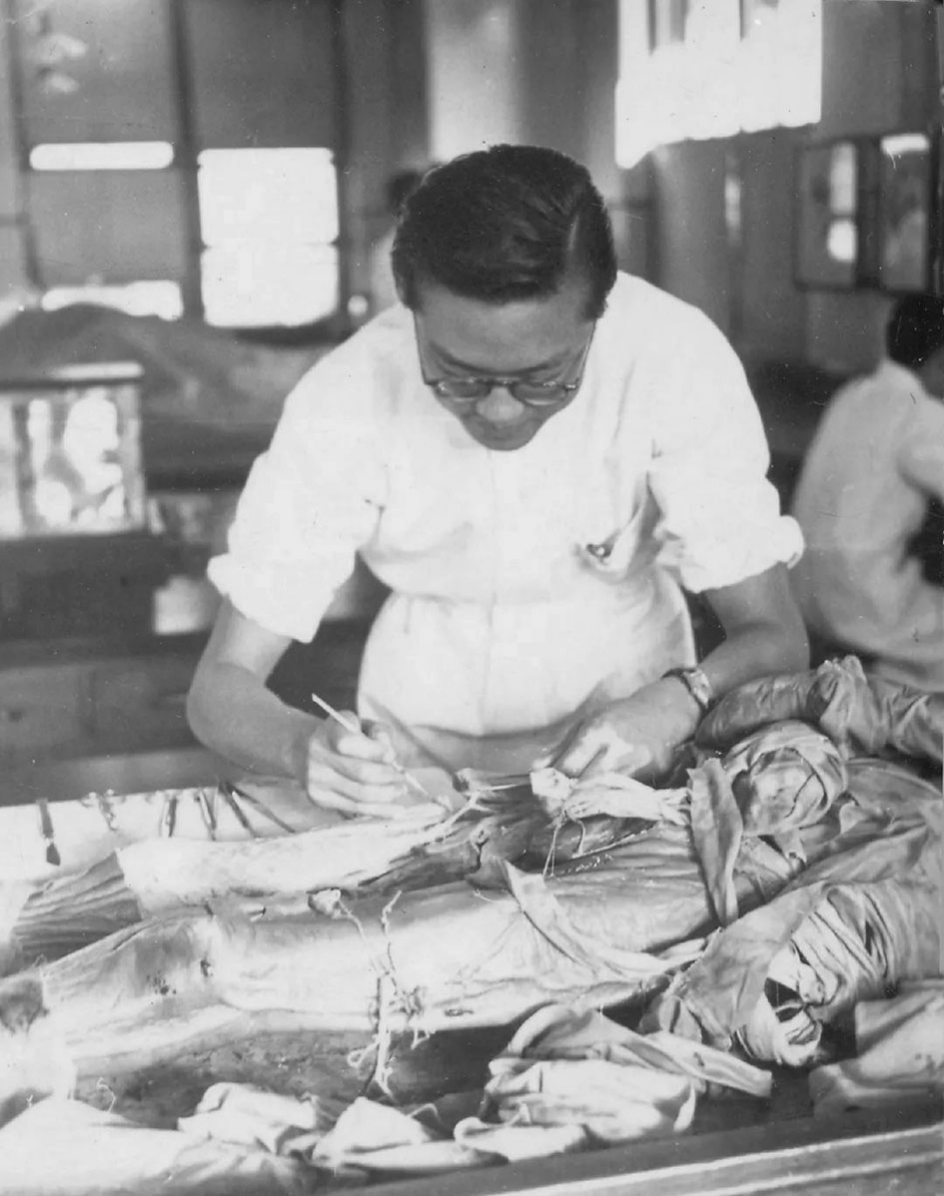
**Qi Fang at Peking Union Medical College in 1941**

Prof. Fang was born in Beijing and studied at Nankai High School in Tianjin from 1931 to 1937. “I witnessed my younger brother’s suffering from hemophilia as a child. He sustained frequent bleeding ever since one year old. The only thing we could do was waiting for the doctor.” This painful experience impelled Prof. Fang to devote himself to medicine. In 1938, Fang was admitted to the Pre-medical Department, Yenching University in Beijing and then entered Peking Union Medical College (PUMC) in 1941. PUMC was compelled to close from early 1942 to 1948 due to the Japanese invasion. During this time, Prof. Fang studied at the Medical School of Saint John’s University in Shanghai, and then West China Union University in Chengdu. In 1946 he received his M.D. degree, earned one of the only two “Golden Key Award”. From 1946 to 1948, he worked at Tianjin Central Hospital. When PUMCH re-opened in 1948, he rejoined it as a chief resident of the Internal Medicine Department (PUMCH, 2018) (Fig. 2).

**Figure 3:**
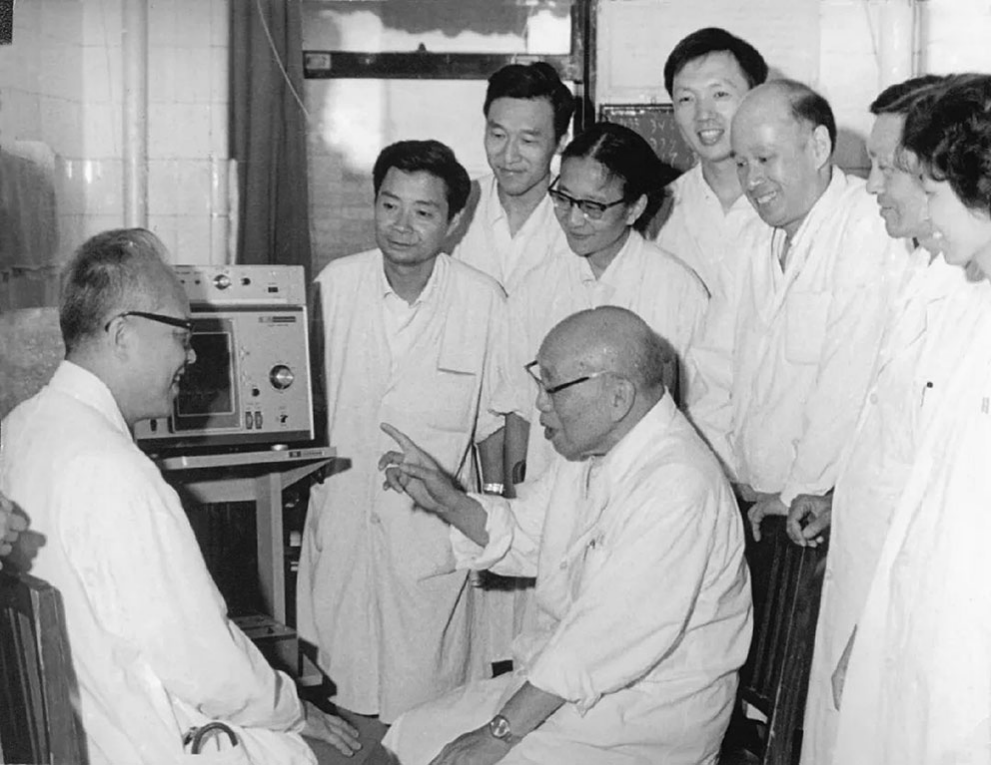
**Prof. Fang working with Prof. Xiaoqian Zhang (张孝骞) and other colleagues from Internal Medicine Department of PUMCH**

One day in 1951, Dr. Fang and his colleague admitted two tuberculosis patients. Expecting learning less new skills and experiences from these common patients, Fang’s colleague sighed and complained. It suddenly struck Dr. Fang that doctors should serve patients unconditionally with best efforts, but not treat them solely as sources of medical practice. If patients serve doctors, doctors would be passionate about rare and challenging cases and apathetic to common and simple cases. Medical practice is not only about curing, but also comforting. Doctors should care and love their patients. Carrying this belief, Dr. Fang successively served as resident, attending physician, associate professor, professor, director of internal medicine, vice president, and honorary president of PUMCH (Wang et al., 2006) (Fig. 3).

Prof. Fang was a founder and pioneer in many areas of cardiology. Since 1950s, he had engaged in the study of various cardiovascular diseases. He early advocated the clinical application of electrocardiography. Together with Prof. Yingkai Wu (吴英恺), Prof. Fang established Fuwai Hospital, China’s first special hospital of cardiovascular diseases in 1956, and was appointed Fuwai Hospital’s first director of Internal Medicine. Prof. Fang led China’s application of cardiac catheterization and pushed forward the diagnosis and treatment of congenital heart diseases. He was also the first to study blood stream dynamics for rheumatic heart diseases and laid the foundation for hemodynamic testing in China. From the 1970s on, Prof. Fang focused on the studies of coronary artery disease, arrhythmia, electrocardiogram and cardiovascular drugs (PUMCH, 2018).

**Figure 4:**
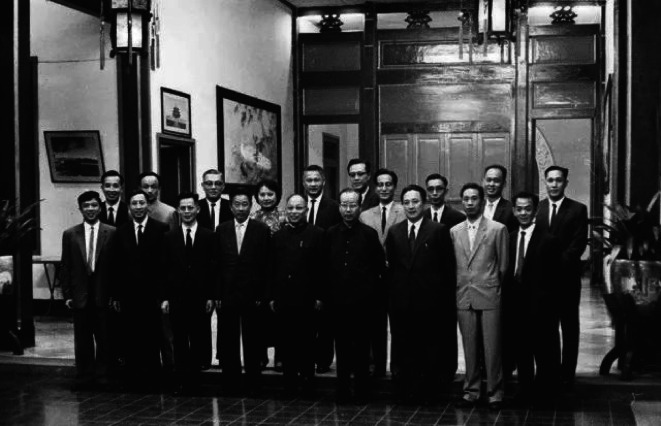
**In 1962, Chinese Medical Team at Chinese Embassy in Indonesia during their consultations for President Sukarno. The sixth from the left in the second row is Prof. Fang**

Prof. Fang became a member of medical team for state leaders in 1956. Since then, he attended many oversea medical tasks for foreign state leaders. From 1962 to 1965, he went to Indonesia five times to attend consultations for President Sukarno. In 1974, Prince Souvanna Phouma of Laos had a heart attack. Vientiane called for urgent help from Beijing, Washington, Moscow, Paris, Bangkok and Manila. Prof. Fang flew to Vientiane overnight under Premier Enlai Zhou’s command and contributed the most effective emergency treatment for Prince Souvanna Phouma with doctors from seven other countries (Guang, 2001) (Fig. 4).

Prof. Fang painstakingly educated young doctors all through his life. He was one of the first group of doctoral tutors of PUMCH. He was chief editor of four books, one of which *Modern Internal Medicine* won the first prize of National Excellent Science and Technology Books Award and second prize of National Science and Technology Progress Award. He gave instructive guidance to medical students and junior doctors with no thought for rewards. Sometimes, they would list Prof. Fang’s name on academic literatures which he helped to plot or revise. However, Prof. Fang always firmly refused (Yan, 1992).

**Figure 5:**
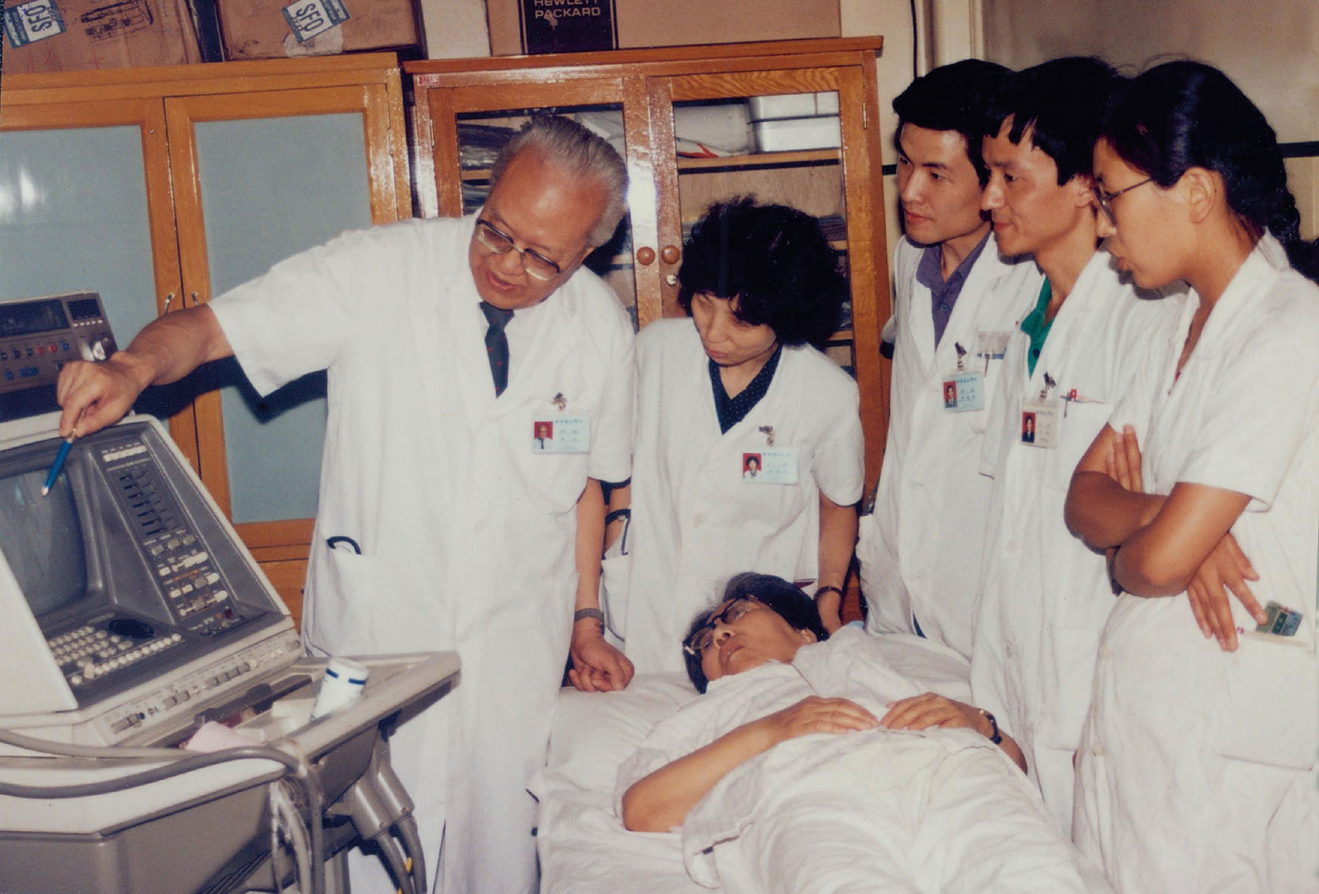
**Prof. Fang (first from the left) guiding Prof. Wenling Zhu (second from the left) and other junior doctors from Cardiology Department of PUMCH in the 1980s**

In an article published in the Chinese Journal of Cardiology in 2018, Prof. Wenling Zhu (朱文玲) from Department of Cardiology, PUMCH wrote: “Prof. Fang paid great attention to academic training for every doctor in our department. After China’s Reform and Opening-up, Prof. Fang immediately started to arrange for our studies abroad. He personally wrote recommendation letters for my study in echocardiography in the US. Prof. Ning Wu (吴宁), Prof. Yuhua Dai (戴玉华), Prof. Baohua Ji (纪宝华) and Prof. Kai You (游凯) all went abroad with his help during the same period. After study, Prof. Wu became the leader in the field of radiofrequency ablation in China. Prof. You became one of the forerunners of Chinese clinical trial of new drug development. And I led China’s clinical study of modern echocardiography. Supported by Prof. Fang, we established the Cardiac Catheterization Laboratory, as well as Molecular Biology Laboratory in PUMCH. Prof. Fang had guided and inspired younger generations to get somewhere (Zhu, 2018) (Fig. 5).

Prof. Fang enthusiastically participated in the work of academic exchange activities and medical associations. He was vice president of the Chinese Medical Association (CMA), director of the Association’s Society of Internal Medicine, and editor-in-chief of the *Chinese Journal of Cardiology*. From 1977 to 1980, he served as a member of the World Health Organization Medical Study Advisory Committee. For three terms he was deputy director of the Society of Cardiology, CMA. He supported the establishment of China Hypertension League (CHL), promoted CHL joining World Hypertension League (PUMCH, 2018).

**Figure 6:**
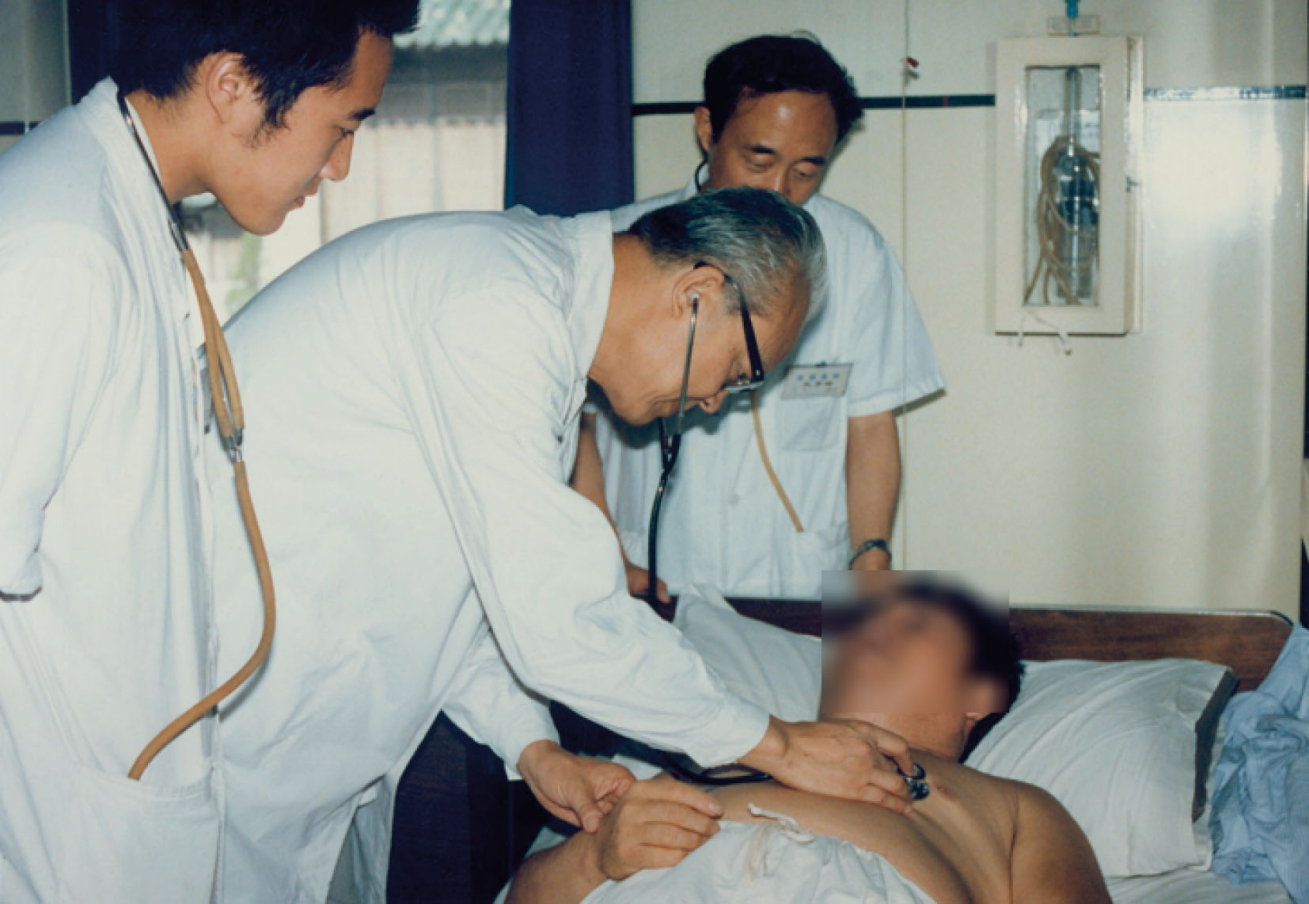
**Prof. Fang examining the patient**

Despite his fame, many titles and positions, putting on the doctor’s white garment, Prof. Fang had nothing but his patients in mind. Once he had kept working 16 h to rescue a severe sepsis patient. Just when he was about to take a break, the ward admitted a little girl with rheumatic heart disease at death’s door. Hearing about the girl’s severe heart failure, Prof. Fang immediately got up and went back to rescue her. In the next 3 days, he kept observing patient recovery without leaving the ward. Finally the girl got out of danger and recovered (Guang, 2001). Countless nights and mornings, the dog days of summer and the dead of winter, Prof. Fang was called from sound sleep, dinner table, bringing hope and consolation to patients and their families. One of his most common words to subordinate doctors was: “Please don’t hesitate to call me if there is problem with patients, anytime!” In the winter, he always rubbed his hands to make them warm before palpation. Rich or poor, educated or illiterate, noble or disadvantaged, Prof. Fang treated his patients without discrimination. He strongly cherished the conviction that patients should receive equal treatment from doctors (Dai, 1985) (Fig. 6).

Prof. Fang earned many honors in his 72-year medical career. In 1954, he received the Three-Class Meritorious Medal, Chinese People’s Volunteer Army. In 1985 he received the title of Model Party Member from the Party committees of Beijing Government and the Ministry of Health. In 1986 he received the National May 1st Labor Medal. In 1989 he received the title of National Distinguished Worker. In 1994 he won the Healthcare Special Contribution Award. In 1995 he was given the Bethune Medal, the highest honor that can be granted to a medical worker in China (PUMCH, 2018).

However, the most cherished “prize” to Prof. Fang was from Premier Enlai Zhou. Prof. Fang looked after Premier Zhou’s health for a long time. In his deathbed and in the presence of Prof. Fang, Premier Zhou asked his wife Yingchao Deng to come close and whispered to her: “Fang is a model Party member…” Prof. Fang joined the Communist Party of China in 1956. After Premier Zhou passed away, Deng gave Prof. Fang a quartz clock. This clock had been sitting on Premier Zhou’s office table till death. The gift meaningfully implied Premier Zhou, as well as Prof. Fang’s extremely busy and tightly scheduled life (Ma, 1997).

On January 30th, 2018, Prof. Fang died of illness at the age of 98. Throughout his selfless life, Prof. Fang took little heed of fame and wealth, and was remembered for his genuine kindness and superb expertise (Chen et al., 2018).
